# Execution by Electricity

**Published:** 1897-08-21

**Authors:** 


### Execution by Electricity.

The manner in which the sentence of death is carried out in many parts of the United States is becoming a matter of serious concern, both from a humanitarian and an international point of view. As is well known, many of the States have legalised the execution of criminals by means of electricity, and to those whose knowledge of electricity is as meagre as their experience of executions, this seems a happy dispatch, a convenient, a painless, and above all, a rapid means of ending life. The good people who have voted for the introduction of this horror have no doubt acted with the best of motives, being influenced by the idea that hanging is the worst of deaths. We will not enter here into the question whether or not a criminal deserves an easy death, but we do state without fear of contradiction that the penalty of death is a very much less severe one than the penalty which so many in the States are now unjustly made to suffer, namely, the penalty of death by torture. The following account of an execution by electricity, taken from the *Medical Record* of New York, will show what we mean, and it is by no means a solitary instance: "An Italian was put to death at Auburn on June 22nd, for the murder of a fellow-countryman eighteen months ago. He was placed in the chair, and five shocks with a current of eighteen hundred and forty volts, each of about one minute duration, were made at intervals. The poor creature was not pronounced dead until eight minutes had elapsed after the first contact was made. The five shocks were necessary because of the imperfect contact of the electrode on the man's leg. The smell of burning flesh was quite distinct in the chamber after the first shock had been given."

What we wish to insist upon is that such a miscarriage of justice as took place in this instance is sure to recur so long as such fanciful methods of execution are made use of. If executions occurred every day no doubt the proportion of mistakes would diminish, but it may be questioned whether any amount of practice will entirely eliminate the chance of error in an experiment like this, an experiment the details of which, from their very nature, cannot be submitted to a preliminary trial. The chances of mistake, however, are infinitely multiplied when the execution has to be performed by men who must be more or less strange to the necessary proceedings, and from want of habituation to the idea of taking life must be under the influence of excitement and agitation.

The preliminary preparations for the rope or for the guillotine are trifling compared with those requisite for the effectual use of electricity as a life-taking agent; the rope, moreover, can be tested and the guillotine can be tried so as to make certain that they are in working order; the short half second which elapses between the falling of the drop and the dislocation of the neck is hardly worth consideration; while as to the guillotine—which, after all, is the most humane form of execution—the time occupied in the severance of the neck is imperceptible. Moreover, both in hanging and beheading there at least is certainty that the man is dead, while in "electrocution" no one seems to know when life or sensibility ceases, and however cleverly the trick may be done in one case it is impossible to ensure that the same conditions shall be present in the next. We repeat that this question has an international bearing. Every civilised nation endeavours to obtain for its subjects justice and fair treatment in whatever part of the world they may be, and we may fairly ask what Italy says to such a torturing of one of her subjects as is described above. When people go into foreign lands they must submit to foreign laws, but there are certain penalties which are outside and beyond the right of any civilised country to impose, and one of them is death by torture.

